# GPU facilitated unsupervised visual feature acquisition

**DOI:** 10.1186/1471-2202-13-S1-P87

**Published:** 2012-07-16

**Authors:** Blake Lemoine, Anthony Maida

**Affiliations:** 1 Center for Advanced Computer Studies, University of Louisiana, Lafayette, LA 70503, USA

## 

This paper examines the effects of parallelizing an unsupervised feature acquisition algorithm using General-Purpose computing on Graphics Processing Units (GPGPU). Previous work in feature learning has established the effectiveness of hierarchical learning methods that refine large quantities of elementary features into small numbers of intermediate-level features. Some of the most effective of these methods alternate between template matching layers that combine features and sub-sampling layers that perform feature scale and/or position invariance. This approach is inspired by the mammalian ventral visual pathway. One such approach that produces highly informative features is a spiking convolutional network trained with STDP-based learning [1]. When using a serial implementation, however, this approach is costly in time and consequently is difficult to scale to a large library of internally complex features. The present paper demonstrates that GPGPU parallelism can be leveraged to overcome the scaling limitations of the serial version. Highly informative features can then be generated in large quantities. By scaling the number of features learned the maximum complexity of learnable classes is significantly increased.
